# Current Awareness on Comparative and Functional Genomics

**DOI:** 10.1002/cfg.57

**Published:** 2001-06


**Comparative and Functional Genomics**
*Comp. Funct. Genom.* 2001; **2:** 187–194
**DOI**:10.1002/cfg.57

### Current awareness on comparative and functional genomics

### 1 Reviews & symposia

2000. Special issue papers: EC funded genome sequencing projects. *DNA Seq* **11:** (3-4)

Banks RE, Dunn MJ, Hochstrasser DF, Sanchez JC, Blackstock W, Pappin DJ, Selby PJ. 2000. St James Univ Hosp, ICRF Canc Med Res Unit, Leeds LS9 7TF, England. Proteomics: New perspectives, new biomedical opportunities. *Lancet* **356:** (9243) 1749.

Bassingthwaighte JB. 2000. Univ Washington, Dept Bioengn, Box 35-7962, Seattle, Wa 98195, USA. Strategies for the physiome project. *Ann Biomed Eng* **28:** (8) 1043.

Bentley DR. 2000. Sanger Ctr, Wellcome Trust Genome Campus, Cambridge CB10 1SA, England. Decoding the human genome sequence (Review). *Hum Mol Genet* **9:** (16) 2353.

Black DL. 2000. UCLA, Howard Hughes Med Inst, 675 Charles E Young Dr Sth, Los Angeles, Ca 90095, USA. Protein diversity from alternative splicing: A challenge for bioinformatics and post-genome biology (Mini-Review). *Cell* **103:** (3) 367.

Black T, Hare R. 2000. Schering Plough Res Inst, Dept Chemotherapy & Mol Genet, 2015 Galloping Hill Rd, Murray Hill, NJ 07974, USA. Will genomics revolutionize antimicrobial drug discovery? *Curr Opin Microbiol* **3:** (5) 522.

Blundell TL, Mizuguchi K. 2000. Univ Cambridge, Dept Biochem, 80 Tennis Court Rd, Cambridge CB2 1QW, England. Structural genomics: An overview. *Prog Biophys Mol Biol* **73:** (5) 289.

Bode J, Schlake T, Iber M, Schubeler D, Seibler J, Snezhkov E, Nikolaev L. 2000. German Ctr Biotechnol Res, RDIF/Epigenetic Regulation, Mascheroder Weg 1, DE-38124 Braunschweig, Germany. The transgeneticist's toolbox: Novel methods for the targeted modification of eukaryotic genomes (Review). *Biol Chem* **381:** (9-10) 801.

Bowman S, Horrocks P. 2000. Sanger Ctr, Pathogen Sequencing Unit, Wellcome Trust Genome Campus, Hinxton CB10 1SA, England. Assessing the impact of *Plasmodium falciparum* genome sequencing (Review). *Microbes Infect* **2:** (12) 1479.

Brookman JL, Denning DW. 2000. Univ Manchester, Sch Biol Sci, 1-800 Stopford Bldg, Oxford Rd, Manchester M13 9PT, England. Molecular genetics in *Aspergillus fumigatus*. *Curr Opin Microbiol* **3:** (5) 468.

Chambers G, Lawrie L, Cash P, Murray GI*. 2000. *Univ Aberdeen, Dept Pathol, Aberdeen AB25 2ZD, Scotland. Proteomics: A new approach to the study of disease (Review). *J Pathol* **192:** (3) 280.

Christendat D, Yee A, Dharamsi A, Kluger Y, Gerstein M, Arrowsmith CH*, Edwards AM. 2000. *Univ Toronto, Ontario Canc Inst, 610 Univ Ave, Toronto, Ontario, Canada M5G 2M9. Structural proteomics: Prospects for high throughput sample preparation. *Prog Biophys Mol Biol* **73:** (5) 339.

Dao N, McCormick PJ, Dewey CF*. 2000. *MIT, Department Mech Engn, 77 Massachusetts Ave, Cambridge, Ma 02139, USA. The human physiome as an information environment. *Ann Biomed Eng* **28:** (8) 1032.

Eisen JA. 2000. Inst Genomic Res, 9712 Med Ctr Dr, Rockville, Md 20850, USA. Assessing evolutionary relationships among microbes from whole-genome analysis. *Curr Opin Microbiol* **3:** (5) 475.

Fraser CM, Eisen J, Fleischmann RD, Ketchum KA, Peterson S. 2000. Inst Genomic Res, 9712 Med Ctr Dr, Rockville, Md 20850, USA. Comparative genomics and understanding of microbiol biology. *Emerg Infect Dis* **6:** (5) 505.

Freeman WM, Robertson DJ, Vrana KE*. 2000. *Wake Forest Univ, Dept Physiol & Pharmacol, Med Ctr Blvd, Winston Salem, NC 27157, USA. Fundamentals of DNA hybridization arrays for gene expression analysis (Review). *Biotechniques* **29:** (5) 1042.

Futcher B. 2000. CSH Lab, POB 10-0, Cold Spring Harbor, NY 11724, USA. Microarrays and cell cycle transcription in yeast (Review). *Curr Opin Cell Biol* **12:** (6) 710.

Gerstein M, Jansen R. 2000. Yale Univ, Dept Mol Biophys & Biochem, 266 Whitney Ave, New Haven, Ct 06520, USA. The current excitement in bioinformatics - Analysis of whole-genome expression data: How does it relate to protein structure and function? *Curr Opin Struct Biol* **10:** (5) 574.

Ghosh D. 2000. Inst Transcription Information, POB 2556, Pittsburgh, Pa 15230, USA. High throughput and global approaches to gene expression (Review). *Comb Chem High Throughout Scr* **3:** (5) 411.

Giegerich R. 2000. Univ Bielefeld, Fac Technol, DE-33615 Bielefeld, Germany. A systematic approach to dynamic programming in bioinformatics (Review). *Bioinformatics* **16:** (8) 665.

Gygi SP, Aebersold R. 2000. Harvard Univ, Dept Cell Biol, Boston, Ma 02115, USA. Mass spectrometry and proteomics. *Curr Opin Chem Biol* **4:** (5) 489.

Haynes PA, Yates JR. 2000. Novartis Agric Discovery Inst, 3115 Merryfield Row, San Diego, Ca 92121, USA. Proteome profiling: Pitfalls and progress (Review). *Yeast* **17:** (2) 81.

Heger A, Holm L*. 2000. *EMBL-EBI, Struct Genomics Grp, Cambridge CB10 1SD, England. Towards a covering set of protein family profiles. *Prog Biophys Mol Biol* **73:** (5) 321.

Heinemann U, Frevert J, Hofmann KP, Illing G, Maurer C, Oschkinat H, Saenger W. 2000. Max Delbruck Ctr Mol Med, Forschungsgrp Kristallog, Robert Rossle Str 10, DE-13122 Berlin, Germany. An integrated approach to structural genomics. *Prog Biophys Mol Biol* **73:** (5) 347.

Holt LJ, Enever C, De Wildt RMT, *Tomlinson IM*. 2000. *MRC Mol Biol Lab, Hills Rd, Cambridge CB2 2QH, England. The use of recombinant antibodies in proteomics (Review). *Curr Opin Biotechnol* **11:** (5) 445.

Jung E, Heller M, Sanchez JC, Hochstrasser DF. 2000. Univ Geneva, Swiss Inst Bioinformat, 1 Michel Servet, CH-1211 Geneva 4, Switzerland. Proteomics meets cell biology: The establishment of subcellular proteomes - Review. *Electrophoresis* **21:** (16) 3369.

Khush RS, Lemaitre B. 2000. CNRS, Ctr Genet Mol, FR-91198 Gif-sur-Yvette, France. Genes that fight infection: What the *Drosophila* genome says about immunity (Review). *Trends Genet* **16:** (10) 442.

Kreutz R. 2000. Free Univ Berlin, Klin Pharmakol Abt, Hindenburg Damm 30, DE-12200 Berlin, Germany. Pharmacogenomics and pharmacogenetics in arterial hypertension (Review). *Dtsch Med Wochenschr* **125:** (46) 1403.

Lambais MR, Goldman MHS, Camargo LEA, Goldman GH. 2000. USP, Escola Super Agr Luiz Queiroz, Avda Padua Dias 11, BR-13418-900 Sao Paulo, Brazil. A genomic approach to the understanding of *Xylella fastidiosa* pathogenicity. *Curr Opin Microbiol* **3:** (5) 459.

Linial M, Yona G. 2000. Hebrew Univ, Inst Life Sci, Dept Biol Chem, IL-91904 Jerusalem, Israel. Methodologies for target selection in structural genomics. *Prog Biophys Mol Biol* **73:** (5) 297.

McDonald WH, Yates JR*. 2000. *Scripps Clin & Res Inst, Dept Cell Biol, 10550 Nth Torrey Pines Rd, La Jolla, Ca 92037, USA. Proteomic tools for cell biology (Review). *Traffic* **1:** (10) 747.

Nelson KE, Paulsen IT, Heidelberg JF, Fraser CM. 2000. Inst Genomic Res, 9712 Med Ctr Dr, Rockville, Md 20850, USA. Status of genome projects for nonpathogenic bacteria and archaea (Review). *Nat Biotechnol* **18:** (10) 1049.

Paterson AH, Bowers JE, Burow MD, Draye X, Elsik CG, Jiang CX, Katsar CS, Lan TH, Lin YR, Ming RG, Wright RJ. 2000. Univ Geor-

In order to keep subscribers up-to-date with the latest developments in their field, this current awareness service is provided by John Wiley & Sons and contains newly-published material on comparative and functional genomics. Each bibliography is divided into 16 sections. 1 Reviews & symposia; 2 General; 3 Large-scale sequencing and mapping; 4 Genome evolution; 5 Comparative genomics; 6 Pathways, gene families and regulons; 7 Pharmacogenomics; 8 Large-scale mutagenesis programmes; 9 Functional genomics; 10 Transcriptomics; 11 Proteomics; 12 Protein structural genomics; 13 Metabolomics; 14 Genomic approaches to development; 15 Technological advances; 16 Bioinformatics. Within each section, articles are listed in alphabetical order with respect to author. If, in the preceding period, no publications are located relevant to any one of these headings, that section will be omitted.

Copyright © 2001 John Wiley & Sons, Ltd.

gia, Appl Genet Technol Ctr, Athens, Ga 30602, USA. Comparative genomics of plant chromosomes. *Plant Cell* **12:** (9) 1523.

Pereira A. 2000. Plant Res Int, POB 16, NL-6700 AA Wageningen, The Netherlands. A transgenic perspective on plant functional genomics (Review). *Transgenic Res* **9:** (4-5) 245.

Riley M, Serres MH. 2000. Marine Biol Lab, Josephine Bay Paul Ctr Comp Mol Biol & Evolut, Woods Hole, Ma 02543, USA. Interim report on genomics of *Escherichia coli* (Review). *Annu Rev Microbiol* **54:** 341.

Rokas A, Holland PWH. 2000. Univ Edinburgh, Inst Cell Anim & Populat Biol, Kings Bldg, West Mains Rd, Edinburgh EH9 3JT, Scotland. Rare genomic changes as a tool for phylogenetics (Review). *Trends Ecol Evolut* **15:** (11) 454.

Sanchez-Carbayo MS, Bornmann W, Cordon-Cardo C. 2000. Mem Sloan Kettering Canc Ctr, Div Mol Pathol, 1275 York Ave, New York, NY 10021, USA. DNA microchips: Technical and practical considerations. *Curr Org Chem* **4:** (9) 945.

Sciochetti SA, Piggot PJ*. 2000. *Temple University, Department Microbiol & Immunol, 3400 Nth Broad St, Philadelphia, Pa 19140, USA. A tale of two genomes: resolution of dimeric chromosomes in *Escherichia coli* and *Bacillus subtilis* (Mini-review). *Res Microbiol* **151:** (7) 503.

Shea JE, Santangelo JD, Feldman RG. 2000. Microsci Ltd, 545 Eskdale Rd, Wokingham RG41 5TU, England. Signature-tagged mutagenesis in the identification of virulence genes in pathogens. *Curr Opin Microbiol* **3:** (5) 451.

Van Wijk KJ. 2000. Univ Stockholm, Arrhenius Labs Nat Sci, Dept Biochem, SE-10691 Stockholm, Sweden. Proteomics of the chloroplast: Experimentation and prediction (Review). *Trends Plant Sci* **5:** (10) 420.

Vivares CP, Metenier G. 2000. Univ Clermont Ferrand, UMR CNRS 6023, Lab Parasitol Mol & Cellulaire, FR-63177 Aubiere, France. Towards the minimal eukaryotic parasitic genome. *Curr Opin Microbiol* **3:** (5) 463.

Walhout AJM, Boulton SJ, Vidal M*. 2000. *Harvard Med Sch, Dept Genet, 44 Binney St, Boston, Ma 02115, USA. Yeast two-hybrid systems and protein interaction mapping projects for yeast and worm (Review). *Yeast* **17:** (2) 88.

Wise RP. 2000. Iowa State Univ, USDA/ARS, Corn Insects & Crop Genet Res Unit, Ames, Ia 50011, USA. Disease resistance: What's brewing in barley genomics. *Plant Dis* **84:** (11) 1160.

Yokoyama S, Matsuo Y, Hirota H, Kigawa T, Shirouzu M, Kuroda Y, Kurumizaka H, Kawaguchi S, Ito Y, Shibata T, Kainosho M, Nishimura Y, Inoue Y, Kuramitsu S. 2000. RIKEN, Genomic Sci Ctr, Tsurumi ku, 1-7-22 Suehiro cho, Yokohama, Kanagawa 230 004, Japan. Structural genomics projects in Japan. *Prog Biophys Mol Biol* **73:** (5) 363.

### 2 General

Gull K. 2000. Univ Manchester, Sch Biol Sci, 2-205 Stopford Bldg, Oxford Rd, Manchester M13 9PT, England. Genomics and post-genomics in parasitology: Genome babble or a real opportunity? *Biochem Soc Trans* **28:** (5) 541.

Hamadeh H, Afshari CA. 2000. NIEHS, Mol Carcinogenesis Lab, POB 12233, Res Triangle Park, NC 27709, USA. Gene chips and functional genomics: A new technology will allow environmental health scientists to track the expression of thousands of genes in a single, fast and easy test. *Am Sci* **88:** (6) 508.

Long H, Aldredge T, Hebert A, Perrin S, Smith M, August P, Newcomb R, Call K. 2000. Aventis, Cambridge Genomics Ctr, Landsdowne St, Cambridge, Ma 02139, USA. Automation technology applications in a genomics center. *Am Lab* **32:** (20) 30.

Miranda LP, Alewood PF. 2000. Carlsberg Lab, Dept Chem, Gamle Carslberg Vej 10, DK-2500 Copenhagen, Denmark. Challenges for protein chemical synthesis in the XXIst century: Bridging genomics and proteomics. *Biopolymers* **55:** (3) 217.

Schwartz I. 2000. NY Med Coll, Dept Biochem & Mol Biol, Valhalla, NY 10595, USA. Microbial genomics: From sequence to function. *Emerg Infect Dis* **6:** (5) 493.

Weinstock GM. 2000. UT Med Sch, Dept Microbiol & Mol Genet, 6431 Fannin, Houston, Tx 77030, USA. Genomics and bacterial pathogenesis. *Emerg Infect Dis* **6:** (5) 496.

### 3 Large-scale sequencing and mapping

Arabidopsis Genome Initiative. 2000. Analysis of the genome sequence of the flowering plant *Arabidopsis thaliana*. *Nature* **408:** (6814) 796.

Burset M, Seledtsov IA, Solovyev VV*. 2000. *Sanger Ctr, Informatic Div, Cambridge CB10 1SA, England. Analysis of canonical and non-canonical splice sites in mammalian genomes. *Nucleic Acids Res* **28:** (21) 4364.

De Villiers EP, Brayton KA, Zweygarth E, Allsopp BA. 2000. Univ Utrecht, Div Parasitol & Trop Vet Med, POB 80-165, NL-3508 TD Utrecht, The Netherlands. Genome size and genetic map of *Cowdria ruminantium*. *Microbiology* **146:** (10) 2627.

Dube S, Dolcini G, Abbott L, Mehta S, Dube D, Gutierrez S, Ceriani C, Esteban E, Ferrer J, Poiesz B*. 2000. *SUNY Upstate Med Univ, Dept Med, 750 East Adams St, Syracuse, NY 13210, USA. The complete genomic sequence of a BLV strain from a Holstein cow from Argentina. *Virology* **277:** (2) 379.

Fujibuchi W, Ogata H, Matsuda H, Kanehisa M*. 2000. *Kyoto Univ, Inst Chem Res, Kyoto 611 0011, Japan. Automatic detection of conserved gene clusters in multiple genomes by graph comparison and P-quasi grouping. *Nucleic Acids Res* **28:** (20) 4029.

Glockner G, Rosenthal A, Valentin K. 2000. IMB Jena, Dept Genome Anal, Beutenbergstr 11, DE-07745 Jena, Germany. The structure and gene repertoire of an ancient red algal plastid genome. *J Mol Evol* **51:** (4) 382.

Gosele C, Hong L, Kreitler T, Rossmann M, Hieke B, Gross U, Kramer M, Himmelbauer H, Bihoreau MT, Kwitek-Black AE, Twigger S, Tonellato PJ, Jacob HJ, Schalkwyk LC, Lindpaintner K, Ganten D, Lehrach H, Knochblauch M*. 2000. *Max-Planck-Inst Mol Genet, Ihnestr 73, DE-14195 Berlin, Germany. High-throughput scanning of the rat genome using interspersed repetitive sequence-PCR markers. *Genomics* **69:** (3) 287.

Govan VA, Leat N, Allsopp M, Davison S. 2000. Univ Western Cape, Dept Microbiol, ZA-7535 Bellville, Rep Sth Africa. Analysis of the complete genome sequence of acute bee paralysis virus shows that it belongs to the novel group of insect-infecting RNA virus. *Virology* **277:** (2) 457.

Guigo R, Agarwal P, Abril JF, Burset M, Fickett JW. 2000. Univ Pompeu Fabra, Inst Municipal Invest Med, Grp Rec Informatica Med, ES-08003 Barcelona, Spain. An assessment of gene prediction accuracy in large DNA sequences. *Genome Res* **10:** (10) 1631.

Hashimoto Y, Hayakawa T, Ueno Y, Fujita T, Sano Y, Matsumoto T. 2000. Kyoto Inst Technol, Dept Appl Biol, Sakyo ku, Kyoto 606 8585, Japan. Sequence analysis of the *Plutella xylostella* granulovirus genome. *Virology* **275:** (2) 358.

Heitman J, Casadevall A, Lodge JK, Perfect JR. 1999. Duke Univ, Dept Genet, Res Dr, Durham, NC 27710, USA. The *Cryptococcus neoformans* genome sequencing project. *Mycopathologia* **148:** (1) 1.

Hoyne PR, Edwards LM, Viari A, Maher LJ. 2000. Mayo Clin, Dept Biochem & Mol Biol, Rochester, Mn 55905, USA. Searching genomes for sequences with the potential to form intrastrand triple helices. *J Mol Biol* **302:** (4) 797.

Kropinski AM. 2000. Queen's Univ, Dept Microbiol & Immunol, Kingston, Ontario, Canada K7L 3N6. Sequence of the genome of the temperate, serotype-converting, *Pseudomonas aeruginosa* bacteriophage D3. *J Bacteriol* **182:** (21) 6066.

Kyrpides NC, Ouzounis CA, Iliopoulos I, Vonstein V, Overbeek R. 2000. Integrated Genomics Inc, Chicago Technol Pk, 2201 West Campbell Pk Dr, Chicago, Il 60612, USA. Analysis of the *Thermotoga maritima* genome combining a variety of sequence similarity and genome context tools. *Nucleic Acids Res* **28:** (22) 4573.

Meksem K, Zobrist K, Ruben E, Hyten D, Quanzhou T, Zhang HB, Lightfoot DA. 2000. Sthn Illinois Univ, Dept Plant Sci & Gen Agr, Room 176, Carbondale, Il 62901, USA. Two large-insert soybean genomic libraries constructed in a binary vector: Applications in chromosome walking and genome wide physical mapping. *Theor Appl Genet* **101:** (5-6) 747.

Ng WV, Kennedy SP, Mahairas GG, Berquist B, Pan M, Shukla HD, Lasky SR, Baliga NS, Thorsson V, Sbrogna J *et al*. 2000. c/o Dassarma S, Univ Massachusetts, Dept Microbiol, Amherst, Ma 01003, USA. Genome sequence of *Halobacterium* species NRC-1. *Proc Natl Acad Sci U S A* **97:** (22) 12176.

Peng J, Korol AB, Fahima T, Roder MS, Ronin YI, Li YC, Nevo E*. 2000. *Inst Plant Genet & Crop Plant Res, DE-06466 Gatersleben,

Copyright © 2001 John Wiley & Sons, Ltd. 

Germany. Molecular genetic maps in wild emmer wheat, *Triticum dicoccoides*: Genome-wide coverage, massive negative interference, and putative quasi-linkage. *Genome Res* **10:** (10) 1509.
Salanoubat M *et al*. 2000. Genoscope/CNRS FRE2231, 2 rue G Cremieux, FR-91057 Evry, France. Sequence and analysis of chromosome 3 of the plant *Arabidopsis thaliana*. *Nature* **408:** (6814) 820.
Santucci A, Trabalzini L, Bovalini L, Ferro E, Neri P, Martelli P. 2000. Univ Siena, Dept Biol Mol, via Fiorentina 1, IT-53100 Siena, Italy. Differences between predicted and observed sequences in *Saccharomyces cerevisiae*. *Electrophoresis* **21:** (17) 3717.
Tabata S *et al*. 2000. c/o Bevan M, John Innes Ctr, Colney Lane, Norwich NR4 7UH, England. Sequence and analysis of chromosome 5 of the plant *Arabidopsis thaliana*. *Nature* **408:** (6814) 823.
Takami H, Nakasone K, Takaki Y, Maeno G, Sasaki R, Masui N, Fuji F, Hirama C, Nakamura Y, Ogasawara N, Kuhara S, Horikoshi K. 2000. Japan Marine Sci & Technol Ctr, Deep Sea Microorganisms Res Grp, 2-15 Natsushima, Kanagawa 237 0061, Japan. Complete genome sequence of the alkaliphilic bacterium *Halodurans* and genomic sequence comparison with *Bacillus subtilis*. *Nucleic Acids Res* **28:** (21) 4317.
Theologis A *et al*. 2000. Univ Calif Berkeley/USDA, Plant Gene Expression Ctr, 800 Buchanan St, Albany, NY 94710, USA. Sequence and analysis of chromosome 1 of the *Arabidopsis thaliana*. *Nature* **408:** (6814) 816.
Tolou H, Couissinier Paris P, Mercier V, Pisano MR, De Lamballerie X, De Micco P, Durand JP. 2000. Inst Trop Med, Unite Virol Trop, Marseille, France. Complete genomic sequence of a dengue Type 2 virus from the French West Indies. *Biochem Biophys Res Commun* **277:** (1) 89.
Upton C, Hogg D, Perrin D, Boone M, Harris NL. 2000. Univ Victoria, Dept Biochem & Microbiol, 150 Petch Bldg, Victoria, Brit Columbia, Canada V8W 3P6. Viral genome organizer: A system for analyzing complete viral genomes. *Virus Res* **70:** (1-2) 55.
Van der Byl C, Kropinski AM*. 2000. *Queens Univ, Dept Microbiol & Immunol, Kingston, Ontario, Canada K7L 3N6. Sequence of the genome of *Salmonella* bacteriophage P22. *J Bacteriol* **182:** (22) 6472.

### 4 Genome evolution

Bailey KA, Pereira SL, Widom J, Reeve JN*. 2000. *Ohio State Univ, Dept Microbiol, 484 West 12th Ave, Columbus, Oh 43210, USA. Archaeal histone selection of nucleosome positioning sequences and the procaryotic origin of histone-dependent genome evolution. *J Mol Biol* **303:** (1) 25.
Barkman TJ, Chenery G, NcNeal JR, Lyons-Weiler J, Ellisens WJ, Moore G, Wolfe AD, De Pamphilis CW. 2000. Western Michigan Univ, Dept Biol Sci, Kalamazoo, Mi 49008, USA. Independent and combined analyses of sequences from all three genomic compartments converge on the root of flowering plant phylogeny. *Proc Natl Acad Sci U S A* **97:** (24) 13166.
Demattei MV, Auge-Gouillou C, Pollet N, Hamelin MH, Meunier-Rotival M, Bigot Y*. 2000. *Fac Sci, CNRS UPRESA 6035, IRBI, Grp Etud Parasites Mol, Parc Grandmont, FR-37200 Tours, France. Features of the *mammal mar1* transposons in the human, sheep, cow and mouse genomes and implications for their evolution. *Mamm Genome* **11:** (12) 1111.
Kiewitz C, Larbig K, Klockgether J, Weinel C, Tummler B*. 2000. Hannover Med Sch, Klin Forschergrp, Zentrum Biochem, Carl Neuberg Str 1, DE-30623 Hannover, Germany. Monitoring genome evolution *ex vivo*: reversible chromosomal integration of a 106 kb plasmid at two $tRNA^{Lys}$ gene loci in sequential *Pseudomonas aeruginosa* airway isolates. *Microbiology* **146:** (10) 2365.
Kirik A, Salomon S, Puchta H*. 2000. *Inst Pflanzengenet & Kulturpflanzenforsch, Corrensstr 3, DE-06466 Gatersleben, Germany. Species-specific double-strand break repair and genome evolution in plants. *EMBO J* **19:** (20) 5562.
Prokhortchouk AV, Ruzov AS. 2000. Russian Acad Sci, Inst Gene Biol, RU-117334 Moscow, Russia. Genome methylation and its role in the functioning of eukaryotic organisms. *Russ J Genet* **36:** (11) 1239.
Ruvinsky I, Silver LM, Gibson-Brown JJ*. 2000. *Washington Univ, Dept Biol, 1 Brookings Dr, St Louis, Mo 63130, USA. Phylogenetic analysis of T-box genes demonstrates the importance of *Amphioxus* for understanding evolution of the vertebrate genome. *Genetics* **156:** (3) 1249.
Vision TJ, Brown DG, Tanksley SD. 2000. USDA/ARS, Ctr Agr Bioinformat, 604 Rhodes Hall, Ithaca, NY 14853, USA. The origins of genomic duplications in *Arabidopsis*. *Science* **290:** (5499) 2114.
Williams EJB, *Hurst LD. 2000. *Univ Bath, Dept Biol & Biochem, Bath BA2 7AY, England. The proteins of linked genes evolve at similar rates. *Nature* **407:** (6806) 900.
Yanai I, Camacho CJ*, Delisi C. 2000. *Boston Univ, Dept Biomed Engn, Boston, Ma 02215, USA. Predictions of gene family distributions in microbial genomes: Evolution by gene duplication and modification. *Phys Rev Lett* **85:** (12) 2641.

### 5 Comparative genomics

Brosch R, Gordon SV, Buchrieser C, Pym AS, Garnier T, Cole ST*. 2000. *Inst Pasteur, Unit Genet Molec Bacterienne, 28 rue Dr Roux, FR-75724 Paris 15, France. Comparative genomics uncovers large tandem chromosomal duplications in *Mycobacterium bovis* BCG Pasteur. *Yeast* **17:** (2) 111.
Brown NF, Beacham IR*. 2000. *Griffith Univ, Sch Hlth Sci, Gold Coast Campus, Southport, Qld 4217, Australia. Cloning and analysis of genomic differences unique to *Burkholderia pseudomallei* by comparison with *B. thailandensis*. *J Med Microbiol* **49:** (11) 993.
Desiere F, Pridmore RD, Brossow H*. 2000. *Nestle Res Ctr, Vers Chez Les Blanc, CH-1000 Lausanne 26, Switzerland. Comparative genomics of the late gene cluster from *Lactobacillus* phages. *Virology* **275:** (2) 294.
Dhillon J, Cowley JA, Wang YH, Walker PJ*. 2000. *CSIRO, Trop Agr, PMB 3, Indooroopilly, Qld 4068, Australia. RNA polymerase (L) gene and genome terminal sequences of ephemeroviruses bovine ephemeral fever virus and Adelaide River virus indicate a close relationship to vesiculoviruses. *Virus Res* **70:** (1-2) 87.
Nikolaichik YA, Donachie WD*. 2000. *Univ Edinburgh, Inst Cell & Mol Biol, Darwin Bldg, Mayfield Rd, Edinburgh EH9 3JR, Scotland. Conservation of gene order amongst cell wall and cell division genes in Eubacteria, and ribosomal genes in Eubacteria and eukaryotic organelles. *Genetica* **108:** (1) 1.
Stinear TP, Jenkin GA, Johnson PDR, Davies JK. 2000. Address not available. Comparative genetic analysis of *Mycobacterium ulcerans* and *Mycobacterium marinum* reveals evidence of recent divergence. *J Bacteriol* **182:** (22) 6322.
Sychrova H, Braun V, Potier S, Souciet JL. 2000. Czech Acad Sci, Dept Membrane Transport, Videnska 1083, CZ-14220 Prague 4, Czech Republic. Organization of specific genomic regions of *Zygosaccharomyces rouxii* and *Pichia sorbitophila*: Comparison with *Saccharomyces cerevisiae*. *Yeast* **16:** (15) 1377.
Wasserman WW, Palumbo M, Thompson W, Fickett JW, Lawrence CE*. 2000. *NY State Dept Hlth, Wadsworth Ctr, Empire State Plaza, POB 509, Albany, NY 12237, USA. Human-mouse genome comparisons to locate regulatory sites. *Nat Genet* **26:** (2) 225.

### 6 Pathways, gene families and regulons

Gross C, Kelleher M, Iyer VR, Brown PO, Winge DR*. 2000. *Univ Utah, Hlth Sci Ctr, Dept Med, Salt Lake City, Ut 84132, USA. Identification of the copper regulon in *Saccharomyces cerevisiae* by DNA microarrays. *J Biol Chem* **275:** (41) 32310.
Juszczuk M, Paczkowska E, Sadowy E, Zagorski W, Hulanicka DM*. 2000. Polish Acad Sci, Inst Biochem & Biofizyki, ul Pawinskiego 5A, PL-02106 Warsaw, Poland. Effect of genomic and subgenomic leader sequences of potato leafroll virus on gene expression. *FEBS Lett* **484:** (1) 33.
Kopp A, Duncan I, Carroll SB*. 2000. *Univ Wisconsin, Howard Hughes Med Inst, 1525 Linden Dr, Madison, Wi 53706, USA. Genetic control and evolution of sexually dimorphic characters in *Drosophila*. *Nature* **408:** (6812) 553.
Lange BM, Rujan T, Martin W, Croteau R*. 2000. *Washington State Univ, Inst Biol Chem, Pullman Wa 99164, USA. Isoprenoid biosynthesis: The evolution of two ancient and distinct pathways across genomes. *Proc Natl Acad Sci U S A* **97:** (24) 13172.
Makarova KS, Aravind L, Daly MJ, Koonin EV. 2000. Uniformed Serv Univ Hlth Sci, Bethesda, Md 20814, USA. Specific expansion of protein families in the radioresistant bacterium *Deinococcus radiodurans*. *Genetica* **108:** (1) 25.

Copyright © 2001 John Wiley & Sons, Ltd. 

McGuire AM, Church GM*. 2000. *Harvard Univ, Dept Genet, 200 Longwood Ave, Boston, Ma 02115, USA. Predicting regulons and their *cis*-regulatory motifs by comparative genomics. *Nucleic Acids Res* **28:** (22) 4523.

### 7 Pharmacogenomics

Adams LD, Geary RL, McManus B, Schwartz SM. 2000. Univ Washington, Dept Pathol, 1959 NE Pacific St, Seattle, Wa 98195, USA. A comparison of aorta and vena cava medial message expression by cDNA array analysis identifies a set of 68 consistently differentially expressed genes, all in aortic media. *Circ Res* **87:** (7) 623.

Chakravarti DN, Fiske MJ, Fletcher LD, Zagursky RJ. 2000. Wyeth Lederle Vaccines, 211 Bailey Rd, West Henrietta, NY 14586, USA. Application of genomics and proteomics for identification of bacterial gene products as potential vaccine candidates. *Vaccine* **19:** (6) 601.

Cummings CA, Relman DA. 2000. Palo Alto VA Hlthcare Syst, Bldg 100, Room D4-123, 3801 Miranda Ave, Palo Alto, Ca 94304, USA. Using DNA microarrays to study host-microbe interactions. *Emerg Infect Dis* **6:** (5) 513.

Dieckgraefe BK, Stenson WF, Korzenik JR, Swanson PE, Harrington CA. 2000. Washington Univ, Div Gastroenterol, 660 Sth Euclid Ave, St Louis, Mo 63110, USA. Analysis of mucosal gene expression in inflammatory bowel disease by parallel oligonucleotide arrays. *Physiol Genomics* **4:** (1) 1.

Dusetti NJ, Tomasini R, Azizi A, Barthet M, Vaccaro MI, Fielder F, Dagorn JC, Iovanna JL. 2000. INSERM U315, 46 Blvd Gaye, FR-13009 Marseille, France. Expression profiling in pancreas during the acute phase of pancreatitis using cDNA microarrays. *Biochem Biophys Res Commun* **277:** (3) 660.

Hough CD, Serman-Baust CA, Pizer ES, Montz FJ, Im DD, Rosenshein NB, Cho KR, Riggins GJ, Morin PJ*. 2000. *NIH/NIA, Gerontol Res Ctr, Biol Chem Lab, 5600 Nathan Shock St, Baltimore, Md 21224, USA. Large-scale serial analysis of gene expression reveals genes differentially expressed in ovarian cancer. *Cancer Res* **60:** (22) 6281.

Irizarry K, Kustanovich V, Li C, Brown N, Nelson S, Wong W, Lee CJ*. 2000. *UCLA, Dept Chem & Biochem, 405 Hilgard Ave, Los Angeles, Ca 90024, USA. Genome-wide analysis of single-nucleotide polymorphisms in human expressed sequences. *Nat Genet* **26:** (2) 233.

Kovarova H, Hajduch M, Korinkova G, Halada P, Krupickova S, Gouldsworthy A, Zhelev N, Strnad M. 2000. Purkyne Med Acad, Inst Immunol & Radiobiol, Trebeska Str 1575, CZ-50001 Hradec Kralove, Czech Republic. Proteomics approach in classifying the biochemical basis of the anticancer activity of the new olomoucine-derived synthetic cyclin-dependent kinase inhibitor, bohemine. *Electrophoresis* **21:** (17) 3757.

Martoglio AM, Tom BDM, Starkey M, Corps AN, Charnock-Jones DS, Smith SK. 2000. Univ Cambridge, Dept Pathol, Tennis Court Rd, Cambridge CB2 1QP, England. Changes in tumorigenesis- and angiogenesis-related gene transcript abundance profiles in ovarian cancer detected by tailored high density cDNA arrays. *Mol Med* **6:** (9) 750.

Niculescu AB, Segal DS, Kuczenski R, Barrett T, Hauger RL, Kelsoe JR. 2000. Univ Calif San Diego, Dept Psychiat, La Jolla, Ca 92093, USA. Identifying a series of candidate genes for mania and psychosis: A convergent functional genomics approach. *Physiol Genomics* **4:** (1) 83.

Ogino T, Wei SW, Wei KC, Moralejo DH, Kose M, Mizuno A, Shima K, Sasaki Y, Yamada T, Matsumoto K*. 2000. *Univ Tokushima, Inst Anim Expt, 3 Kuramoto, Tokushima 770 8503, Japan. Genetic evidence for obesity loci involved in the regulation of body fat distribution in obese Type 2 diabetes rat, OLETF. *Genomics* **70:** (1) 19.

Ono K, Tanaka T, Tsunoda T, Kitahara O, Kihara C, Okamoto A, Ochiai K, Takagi T, Nakamura Y*. 2000. *Univ Tokyo, Ctr Human Genome, Minato ku, 4-6-1 Shirokanedai, Tokyo 108 8639, Japan. Identification by cDNA microarray of genes involved in ovarian carcinogenesis. *Cancer Res* **60:** (18) 5007.

### 8 Large-scale mutagenesis programmes

Hoffman LM, Jendrisak JJ, Meis RJ, Goryshin IY, Reznikoff WS. 2000. EPICTR Technol, 1202 Ann St, Madison, Wi 53713, USA. Transposome insertional mutagenesis and direct sequencing of microbial genomes. *Genetica* **108:** (1) 19.

Koprek T, McElroy D, Louwerse J, Williams-Carrier R, Lemaux PG. 2000. Univ Calif, Dept Plant & Microbial Biol, Berkeley, Ca 94720, USA. An efficient method for dispersing Ds elements in the barley genome as a tool for determining gene function. *Plant J* **24:** (2) 253.

### 9 Functional genomics

Blasco A, Sanz P. 2000. CSIC-Inst Biomed, Jaime Roig 11, ES-46101 Valencia, Spain. Disruption and functional analysis of six ORFs on chromosome IV: *YDL053c*, *YDL072c*, *YDL073w*, *YDL076c*, *YDL077c* and *YDL080c*. *Yeast* **16:** (15) 1437.

Drawid A, Jansen R, Gerstein M*. 2000. *Yale Univ, Dept Mol Biophys & Biochem, 266 Whitney Ave, New Haven, Ct 06520, USA. Genome-wide analysis relating expression level with protein subcellular localization. *Trends Genet* **16:** (10) 426.

Fiehn O, Kopka J, Dormann P, Altmann T, Trethewey RN, Willmitzer L. 2000. Max-Planck-Inst Mol Plant Physiol, DE-14424 Potsdam, Germany. Metabolite profiling for plant functional genomics. *Nat Biotechnol* **18:** (11) 1157.

Fraser AG, Kamath RS, Zipperlen P, Martinez-Campos M, Sohrmann M, Ahringer J*. 2000. *Univ Cambridge, Wellcome CRC Inst, Cambridge CB2 1QR, England. Functional genomic analysis of *C. elegans* chromosome I by systematic RNA interference. *Nature* **408:** (6810) 325.

Friis C, Jensen LJ, Ussery DW*. 2000. *Tech Univ Denmark, Ctr Biol Sequence Anal, Bldg 208, DK-2800 Lyngby, Denmark. Visualization of pathogenicity regions in bacteria. *Genetica* **108:** (1) 47.

Fromont-Racine, Mayes AE, Brunet-Simon A, Rain JC, Colley A, Dix I, Decourty L, Joly N, Ricard F, Beggs JD, Legrain P*. 2000. *Inst Pasteur, CNRS URA 1300, Genet Interact Macromol, 25-28 rue Dr Roux, FR-75724 Paris 15, France. Genome-wide protein interaction screens reveal functional networks involving Sm-like proteins. *Yeast* **17:** (2) 95.

Gonczy P, Echeverri C, Oegema K, Coulson A, Jones SJM, Copley RR, Duperon J, Oegema J, Brehm M, Cassin E *et al*. 2000. Swiss Inst Expt Canc Res, CH-1066 Epalinges, Switzerland. Functional genomic analysis of cell division in *C. elegans* using RNAi of genes on chromosome III. *Nature* **408:** (6810) 331.

Jung MH, Kim SC, Jeon GA, Kim SH, Kim Y, Choi KS, Park SI, Joe MK, Kimm K*. 2000. *Natl Inst Hlth, Dept Biomed Res, Eunpyung ku, 5 Nokbun dong, Seoul 122701, South Korea. Identification of differentially expressed genes in normal and tumor human gastric tissue. *Genomics* **69:** (3) 281.

Khan SA, Zhang N, Ismail T, El-Moghazy AN, Butt A, Wu J, Merlotti C, Hayes A, Gardner DCJ, Oliver SG*. 2000. *Univ Manchester, Sch Biol Sci, 2.205 Stopford Bldg, Oxford Rd, Manchester M13 9PT, England. Functional analysis of eight open reading frames on chromosomes XII and XIV of Saccharomyces cerevisiae. *Yeast* **16:** (16) 1457.

Leikauf GD, McDowell SA, Gammon K, Wesselkamper SC, Bachurski CJ, Puga A, Wiest JS, Leikauf JE*, Prows DR. 2000. *Univ Cincinnati, Dept Environm Hlth, Box 670056, Cincinnati, Oh 45267, USA. Functional genomics of particle-induced lung injury. *Inhal Toxicol* **12:** (Suppl 3) 59.

Myler PJ, Sisk E, McDonagh PD, Martinez-Calvillo S, Schnaufer A, Sunkin SM, Yan S, Madhubala R, Ivens A. 2000. Seattle Biomed Rd Inst, 4 Nickerson St, Seattle, Wa 98109, USA. Genomic organization and gene function in *Leishmania*. *Biochem Soc Trans* **28:** (5) 527.

Paulsen IT, Reizer J, Jin RZ, Lin ECC, Saier MH*. 2000. *Inst Genomic Res, Rockville, Md 20850, USA. Functional genomic studies of dihydroxyacetone utilization in *Escherichia coli*. *Microbiology* **146:** (10) 2343.

Rudy Y. 2000. Case Western Reserve Univ, Cardiac Bioelect Res & Training Ctr, Cleveland, Oh 44106, USA. From genome to physiome: Integrative models of cardiac excitation. *Ann Biomed Eng* **28:** (8) 945.

Schreiber S, Hampe J, Eickhoff H, Lehrach H. 2000. Univ Kiel, Med Klin 1, Schittenhelmstr 12, DE-24105 Kiel, Germany. Functional genomics in gastroenterology. *Gut* **47:** (5) 601.

Sun YH, Bakshi S, Chalmers R, Tang CM*. 2000. *Univ Oxford, John Radcliffe Hosp, Dept Paediat, Oxford OX3 9DU, England. Functional genomics of *Neisseria meningitidis* pathogenesis. *Nat Med* **6:** (11) 1269.

Willer M, Regnacq M, Reid PJ, Tyson JR, Cui W, Wilkinson BM, Stirling CJ*. 2000. *Univ Manchester, Sch Biol Sci, 2.205 Stopford Bldg, Oxford Rd, Manchester M13 9PT, England. Disruption and functional

Copyright © 2001 John Wiley & Sons, Ltd. 

analysis of six ORFs on chromosome XII of *Saccharomyces cerevisiae*: *YLR124W*, *YLR125w*, *YLR126c*, *YLR127c*, *YLR128w* and *YLR129w*. *Yeast* **16:** (15) 1429.

### 10 Transcriptomics

Ablett E, Seaton G, Scott K, Shelton D, Graham MW, Baverstock P, Lee LS, Henry R. 2000. Sthn Cross Univ, Ctr Plant Conservat Genet, POB 157, Lismore, NSW 2480, Australia. Analysis of grape ESTs: Global gene expression patterns in leaf and berry. *Plant Sci* **159:** (1) 87.

Cavalieri D, Townsend JP, Hartl DL*. 2000. *Harvard Univ, Dept Organism & Evolutionary Biol, 16 Divin Ave, Cambridge, Ma 02138, USA. Manifold anomalies in gene expression in a vineyard isolate of *Saccharomyces cerevisiae* revealed by DNA microarray analysis. *Proc Natl Acad Sci U S A* **97:** (22) 12369.

Erwin CR, Falcone RA, Stern LE, Kemp CJ, Warner BW. 2000. Univ Cincinnati, Children's Hosp, Med Ctr, 3333 Burnet Ave, Cincinnati, Oh 45229, USA. Analysis of intestinal adaptation gene expression by cDNA expression arrays. *J Parenter Enter Nutr* **24:** (6) 311.

Goh SH, Park JH, Lee YJ, Lee HG, Yoo HS, Lee IC, Park JH, Kim YS*, Lee CC. 2000. *Korea Res Inst Biosci & Biotechnol, Taejon 305333, South Korea. Gene expression profile and identification of differentially expressed transcripts during human intrathymic T-cell development by cDNA sequencing analysis. *Genomics* **70:** (1) 1.

Guo QM, Malek RL, Kim S, Chiao C, He M, Ruffy M, Sanka K, Lee NH, Dang CV, Liu ET*. 2000. *NCI, Dept Canc & Cell Biol, Mol Signaling & Oncogenesis, Bldg 31, Bethesda, Md 20892, USA. Identification of c-Myc responsive genes using rat cDNA microarray. *Cancer Res* **60:** (21) 5922.

Harmer SL, Hogenesch LB, Straume M, Chang HS, Han B, Zhu T, Wang X, Kreps JA, Kay SA*. 2000. *Scripps Clin, Dept Cell Biol, 10550 Nth Torrey Pines Rd, La Jolla, Ca 92037, USA. Orchestrated transcription of key pathways in *Arabidopsis* by the circadian clock. *Science* **290:** (5499) 2110.

Hill AA, Hunter CP, Tsung BT, Tucker-Kellogg G, Brown EL. 2000. Genet Inst Inc, Dept Genomics, Cambridge, Ma 02140, USA. Genomic analysis of gene expression in *C. elegans*. *Science* **290:** (5492) 809.

Klevecz RR, Dowse HB. 2000. City of Hope Natl Med Ctr, Beckman Res Inst, Dept Biol, 145 East Duarte Rd, Duarte, Ca 991010, USA. Tuning in the transcriptome: Basins of attraction in the yeast cell cycle. *Cell Prolif* **33:** (4) 209.

Lecher P, Petit N, Le Goff S, Alziari S*. 2000. *Univ Clermont Ferrand, CNRS UMR 6547, Equipe Genome Mitochondrial, FR-63177 Aubiere, France. Quantitative analysis, by ultrastructural *in situ* hybridization, of mitochondrial genomes and their expression in mid-gut and ovarian cells of a mutant strain of *Drosophila subobscura*. *Biol Cell* **92:** (5) 341.

Leemans R, Egger B, Loop T, Kammermeier L, He HQ, Hartmann B, Certa U, Hirth F, Reichert H. 2000. Univ Basel, Bioctr, CH-4056 Basel, Switzerland. Quantitative transcript imaging in normal and heat-shocked *Drosophila* embryos by using high-density oligonucleotide arrays. *Proc Natl Acad Sci U S A* **97:** (22) 12138.

Li BR, Chang TF, Larson A, Ding JQ. 2000. Case Western Reserve Univ, Dept Biochem, Wood Basic Sci Bldg, Cleveland, Oh 44106, USA. Identification of mRNAs expressed in tumor-infiltrating lymphocytes by a strategy for rapid and high throughput screening. *Gene* **255:** (2) 273.

Peterson S, Cline RT, Tettelin H, Sharov V, Morrison DA*. 2000. *Univ Illinois, Dept Biol Sci, 900 Sth Ashland Ave, Chicago, Il 60607, USA. Gene expression analysis of the *Streptococcus pneumoniae* competence regulons by use of DNA microarrays. *J Bacteriol* **182:** (21) 6192.

Riechmann JL, Heard J, Martin G, Reuber L, Jiang CZ, Keddie J, Adam L, Pineda O, Ratcliffe OJ, Samaha RR, Creelman R, Pilgrim M, Broun P, Zhang JZ, Ghandehari D, Sherman BK, Yu GL. 2000. Mendel Biotechnol, 21375 Cabot Blvd, Hayward, Ca 94545, USA. *Arabidopsis* transcription factors: Genome-wide comparative analysis among eukaryotes. *Science* **290:** (5499) 2105.

Schenk PM, Kazan K, Wilson I, Anderson JP, Richmond T, Somerville SC, Manners JM*. 2000. *Univ Queensland, Cooperat Res Ctr Trop Plant Pathol, St Lucia, Qld 4072, Australia. Coordinated plant defense responses in *Arabidopsis* revealed by microarray analysis. *Proc Natl Acad Sci U S A* **97:** (21) 11655.

Taegtmeyer H. 2000. UT Med Sch, Div Cardiol, Houston, Tx 77030, USA. Genetics of energetics: Transcriptional responses in cardiac metabolism. *Ann Biomed Eng* **28:** (8) 871.

Thompson BJL, Shang CA, Waters MJ*. 2000. *Univ Queensland, Dept Physiol & Pharmacol, Brisbane, Qld 4072, Australia. Identification of genes induced by growth hormone in rat liver using cDNA arrays. *Endocrinology* **141:** (11) 4321.

Velculescu VE, Vogelstein B, Kinzler KW. 2000. John Hopkins Univ, Oncol Ctr, 1650 Orleans St, Baltimore, Md 21231, USA. Analysing uncharted transcriptomes with SAGE. *Trends Genet* **16:** (10) 423.

Verrills NM, Harry JH, Walsh BJ, Hains PG, Robinson ES. 2000. MacQuarie Univ, APAF, Level 4, Bldg F7B, Sydney, NSW 2109, Australia. Cross-matching marsupial proteins with eutherian mammal databases: Proteome analysis of cells from UV-induced skin tumours of an opossum (*Monodelphis domestica*). *Electrophoresis* **21:** (17) 3810.

### 11 Proteomics

Cahill A, Cunningham CC. 2000. Thomas Jefferson Univ, Dept Pathol Anat & Cell Biol, Philadelphia, Pa 19107, USA. Effects of chronic ethanol feeding on the protein composition of mitochondrial ribosomes. *Electrophoresis* **21:** (16) 3420.

Chen HC, Higgins J, Kondorosi E, Kondorosi A, Djordjevic MA, Weinman JJ, Rolfe BG*. 2000. *Australian Natl Univ, Genomic Interact Grp, Box 475, Canberra, ACT 2601, Australia. Identification of nolR-regulated proteins in *Sinorhizobium meliloti* using proteome analysis. *Electrophoresis* **21:** (17) 3823.

Chen HC, Higgins J, Oresnik IJ, Hynes MF, Natera S, Djordjevic MA, Weinman JJ, Rolfe BG*. 2000. *Address as above. Proteome analysis demonstrates complex replicon and luteolin interactions in pSymacured derivatives of *Sinorhizobium meliloti* strain 2011. *Electrophoresis* **21:** (17) 3833.

Decker G, Wanner G, Zenk MH, Lottspeich F. 2000. Max-Planck-Inst Biochem, Analyt Prot Chem Grp, Klopferspitz 18 A, DE-82152 Martinsried, Germany. Characterization of proteins in latex of the opium poppy (*Papaver somniferum*) using two-dimensional gel electrophoresis and microsequencing. *Electrophoresis* **21:** (16) 3500.

Fivaz M, Vilbois F, Pasquali C, Van der Goot FG*. 2000. *Univ Geneva, Dept Biochem, 30 Quai Ernest Ansermet, CH-1211 Geneva 4, Switzerland. Analysis of glycosyl phosphatidylinositol-anchored proteins by two-dimensional gel electrophoresis. *Electrophoresis* **21:** (16) 3351.

Frey JR, Fountoulakis M, Lefkovits I. 2000. Basel Inst Immunol, Grenzacherstr 487, CH-4005 Basel, Switzerland. Proteome analysis of activated murine B-lymphocytes. *Electrophoresis* **21:** (17) 3730.

Fulda S, Huang F, Nilsson F, Hagemann M, Norling B. 2000. Univ Rostock, Inst Mol Physiol & Biotechnol, Doberaner Str 143, DE-18051 Rostock, Germany. Proteomics of *Synechocystis* sp strain PCC 6803: Identification of periplasmic proteins in cells grown at low and high salt concentrations. *Eur J Biochem* **267:** (19) 5900.

Gao HY, Shen YF, Veenstra TD, Harkewicz R, Anderson GA, Bruce JE, Pasa-Tolic L, Smith RD*. 2000. *Pacific NW Natl Lab, Environm Mol Sci Lab, POB 999, Richland, Wa 99352, USA. Two-dimensional electrophoretic/chromatographic separations combined with electrospray ionization FTICR mass spectrometry for high throughput proteome analysis. *J Microcolumn Sep* **12:** (7) 383.

Hu JC. 2000. Texas A&M Univ, Dept Biochem & Biophys, Biochem Biophys Bldg, College Station, Tx 77843, USA. A guided tour in protein interaction space: Coiled coils from the yeast proteome (Commentary). *Proc Natl Acad Sci U S A* **97:** (24) 12935.

Jefferies JR, Brophy PM, Barrett J. 2000. Univ Wales, Inst Biol Sci, Edward Llwyd Bldg, Aberystwyth SY23 3DA, Wales. Investigation of *Fasciola hepatica* sample preparation for two-dimensional electrophoresis. *Electrophoresis* **21:** (17) 3724.

Journet A, Chapel A, Kieffer S, Louwagie M, Luche S, Garin J. 2000. CEA, Dept Biol Mol & Struct, 17 rue Martyrs, FR-38054 Grenoble 9, France. Towards a human repertoire of monocytic lysosomal proteins. *Electrophoresis* **21:** (16) 3411.

Jung E, Hoogland C, Chiappe D, Sanchez JC, Hochstrasser DF. 2000. Univ Geneva, Swiss Inst Bioinformatics, 1 Michel Servet, CH-1211 Geneva 4, Switzerland. The establishment of a human liver nuclei two-dimensional electrophoresis reference map. *Electrophoresis* **21:** (16) 3483.

Koc EC, Burkhart W, Blackburn K, Moseley A, Koc H, Spremulli LL*. 2000. *Univ Nth Carolina, Dept Chem, CB 3290, Chapel Hill, NC 27599, USA. A proteomics approach to the identification of mammal-

Copyright © 2001 John Wiley & Sons, Ltd. 

ian mitochondrial small subunit ribosomal proteins. *J Biol Chem* **275:** (42) 32585.

Kristoffersen HE, Flengsrud R*. 2000. *Agr Univ Norway, Dept Chem & Biotechnol, POB 5040, NO-1432 As, Norway. Separation and characterization of basic barley seed proteins. *Electrophoresis* **21:** (17) 3693.

Launhardt H, Munder T*. 2000. *Hans Knoll Inst Naturstoff Forsch eV, Dept Cell & Mol Biol, Beutenbergerstr 11, DE-07745 Jena, Germany. Post-translational regulation of *Saccharomyces cerevisiae* proteins tagged with the hormone binding domains of mammalian nuclear receptors. *Mol Gen Genet* **264:** (3) 317.

Liu B, Marks JD*. 2000. *Univ Calif, Dept Anesthesia, 1001 Potrero Ave, San Francisco, Ca 94110, USA. Applying phage antibodies to proteomics: Selecting single chain Fv antibodies to antigens blotted on nitrocellulose. *Anal Biochem* **286:** (1) 119.

Lopez MF, Kristal BS, Chernokalskaya E, Lazarez A, Shestopalov AI, Bogdanova A, Robinson M. 2000. Genomic Solutions, 22 Alpha Rd, Chelmsford, Ma 01824, USA. High-throughput profiling of the mitochondrial proteome using affinity fractionation and automation. *Electrophoresis* **21:** (16) 3427.

Morel V, Poschet R, Traverso V, Deretic D*. 2000. *Univ Michigan, Dept Ophthalmol & Visual Sci, 1000 Wall St, Ann Arbor, Mi 48105, USA. Towards the proteome of the rhodopsin-bearing post-Golgi compartment of retinal photoreactor cells. *Electrophoresis* **21:** (16) 3460.

Nouwens AS, Cordwell SJ, Larsen MR, Molloy MP, Gillings M, Willcox MDP, Walsh BJ. 2000. MacQuarie Univ, APAF, Level 4, Bldg F7B, Sydney, NSW 2109, Australia. Complementing genomics with proteomics: The membrane subproteome of *Pseudomonas aeruginosa* PAO1. *Electrophoresis* **21:** (17) 3797.

Ohlmeier S, Scharf C, Hecker M*. 2000. *Univ Greifswald, Inst Mikrobiol & Mol Biol, Friedrich Ludwig Jahn Str 15, DE-17487 Greifswald, Germany. Alkaline proteins of *Bacillus subtilis*: First steps towards a two-dimensional alkaline master gel. *Electrophoresis* **21:** (17) 3701.

Pardo M, Ward M, Bains S, Molina M, Blackstock W, Gil C*, Nombela C. 2000. *Univ Complutense Madrid, Dept Microbiol II, ES-28040 Madrid, Spain. A proteomic approach for the study of *Saccharomyces cerevisiae* cell wall biogenesis. *Electrophoresis* **21:** (16) 3396.

Prime TA, Sherrier DJ, Mahon P, Packman LC, Dupree P*. 2000. *Dept Biochem, Plant Cell Biol Lab, Downing Site, Cambridge CB2 1QW, England. A proteomic analysis of organelles *Arabidopsis thaliana*. *Electrophoresis* **21:** (16) 3488.

Regula JT, Ueberle B, Boguth G, Gorg A, Schnolzer M, Herrmann R*, Frank R. 2000. *Univ Heidelberg, Zentrum Mol Biol, Neuenheimer Feld 282, DE-69120 Heidelberg, Germany. Towards a two-dimensional proteome map of *Mycoplasma pneumoniae*. *Electrophoresis* **21:** (17) 3765.

Rosenkrands I, King A, Weldingh K, Moniatte M, Moertz E, Andersen P. 2000. Statens Serum Inst, Dept TB Immunol, 5 Artillerivej, DK-2300 Copenhagen, Denmark. Towards the proteome of *Mycobacterium tuberculosis*. *Electrophoresis* **21:** (17) 3740.

Santoni V, Kieffer S, Desclaux D, Masson F, Rabilloud T. 2000. Ecole Natl Super Agron, CNRS/UMR 5004, INRA, Pl Viala, FR-34060 Montpellier 1, France. Membrane proteomics: Use of additive main effects with multiplicative interaction model to classify plasma membrane proteins according to their solubility and electrophoretic properties. *Electrophoresis* **21:** (16) 3329.

Skylas DJ, Mackintosh JA, Cordwell SJ, Basseal DJ, Walsh BJ, Harry J, Blumenthal C, Copeland L, Wrigley CW, Rathmell W. 2000. MacQuarie Univ, Australian Proteome Anal Facil, North Ryde, NSW 2109, Australia. Proteome approach to the characterisation of protein composition in the developing and mature wheat-grain endosperm. *J Cereal Sci* **32:** (2) 169.

Taylor RS, Wu CC, Hays LG, Eng JK, Yates JR, Howell KE*. 2000. *Univ Colorado, Dept Cellular & Struct Biol, 4200 East 9th Ave, Denver, Co 80262, USA. Proteomics of rat liver Golgi complex: Minor proteins are identified through sequential fractionation. *Electrophoresis* **21:** (16) 3441.

Toda T. 2000. Tokyo Metropolitan Inst Gerontol, Dept Gene Regulat & Prot Funct, Itabashi ku, 35-2 Sakaecho, Tokyo 173 0015, Japan. Current status and perspectives of proteomics in aging research. *Exp Gerontol* **35:** (6-7) 803.

Topbas OF, Jehle R, Sinha P, Rustow B*. 2000. *Humboldt Univ, Univ Hosp Charite, Dept Neonatol, Schumannstr 20-21, DE-10098 Berlin, Germany. An electrophoretic study of vitamin E status and expression of heat shock proteins in alveolar type II and liver cells. *Electrophoresis* **21:** (17) 3552.

Vallorani L, Bernardini F, Sacconi C, Pierleoni R, Pieretti B, Piccoli G, Buffalini M, Stocchi V*. 2000. *Univ Urbino, Ist Chim Biol Giorgio Fornaini, via A Saffi 2, IT-61029 Urbino, PU, Italy. Identification of *Tuber borchii* Vittad. mycelium proteins separated by two-dimensional polyacrylamide gel electrophoresis using amino acid analysis and sequence tagging. *Electrophoresis* **21:** (17) 3710.

Verma R, Chen S, Feldman R, Schieltz D, Yates J, Dohmen T, Deshaies RJ. 2000. Novartis Agr Discovery Inst, 3115 Merryfield Row, San Diego, Ca 92130, USA. Proteasomal proteomics: Identification of nucleotide-sensitive proteasome-interacting proteins by mass spectrometric analysis of affinity-purified proteasomes. *Mol Biol Cell* **11:** (10) 3425.

Wu CC, Howell KE*, Neville MC, Yates JR, McManaman JL. 2000. *Univ Colorado, Dept Cellular & Struct Biol, 4200 East 9th Ave, Denver, Co 80262, USA. Proteomics reveal a link between the endoplasmic reticulum and lipid secretory mechanisms in mammary epithelial cells. *Electrophoresis* **21:** (16) 3470.

Wu CC, Yates JR, Neville MC, Howell KE*. 2000. *Univ Colorado, Dept Cellular & Struct Biol, 4200 East 9th Ave, Denver, Co 80262, USA. Proteomic analysis of two functional states of the Golgi complex in mammary epithelial cells. *Traffic* **1:** (10) 769.

Zhu H, Klemic JF, Chang S, Bertone P, Casamayor A, Klemic KG, Smith D, Gerstein M, Reed MA, Snyder M*. 2000. *Yale Univ, Dept Mol Cellular & Dev Biol, New Haven, Ct 06520, USA. Analysis of yeast protein kinases using protein chips. *Nat Genet* **26:** (3) 283.

### 12 Protein structural genomics

Abergel C, Monchois V, Chenivesse S, Jeudy S, Claverie JM. 2000. Aventis, CNRS UMR1889, 31 Chemin Joseph Aiguier, FR-13402 Marseille 20, France. Crystallization and preliminary crystallographic study of b0020 and 'ORFan' protein of unknown function from *Escherichia coli*. *Acta Crystallogr D Biol Crystallogr* **56:** (12) 1694.

Christendat D, Yee A, Dharamsi A, Kluger Y, Savchenko A, Cort JR, Booth V, Mackereth CD, Saridakis V, Ekiel I, Kozlov G, Maxwell KL, Wu N, McIntosh LP, Gehring K, Kennedy MA, Davidson AR, Pai EF, Gerstein M, Edwards AM, Arrowsmith CH*. 2000. *Univ Toronto, Ontario Canc Inst, 610 Univ Ave, Toronto, Ontario, Canada M5G 2M9. Structural proteomics of an archaeon. *Nat Struct Biol* **7:** (10) 903.

Singer GAC, Hickey DA*. 2000. *Univ Ottawa, Dept Biol, 30 Marie Curie St, Ottawa, Ontario, Canada K1N 6N5. Nucleotide bias causes a genomewide bias in the amino acid composition of proteins. *Mol Biol Evol* **17:** (11) 1581.

Wang YL, Bryant S, Tatusov R, Tatusova T*. 2000. *NIH/Natl Lib Med, Natl Ctr Biotechnol Information, Bethesda, Md 20894, USA. Links from genome proteins to known 3-D structures. *Genome Res* **10:** (10) 1643.

### 14 Genomic approaches to development

Lyko F, Ramsahoye BH, Jaenisch R. 2000. Whitehead Inst Biomed Res, 9 Cambridge Ctr, Cambridge, Ma 02142, USA. DNA methylation in *Drosophila melanogaster*. *Nature* **408:** (6812) 538.

Neidhardt L, Gasca S, Wertz K, Obermayr F, Worpenberg S, Lehrach H, Herrmann BG*. 2000. *Max-Planck-Inst Immunbiol, Abt Entwicklungsbiol, Stubeweg 51, DE-79108 Freiburg, Germany. Large-scale screen for genes controlling mammalian embryogenesis, using high-throughput gene expression analysis in mouse embryos. *Mech Dev* **98:** (1-2) 77.

Wertz K, Herrmann BG*. 2000. *Address as above. Large-scale screen for genes involved in gonad development. *Mech Dev* **98:** (1-2) 51.

Zou S, Meadows S, Sharp L, Jan LY, Jan YN*. 2000. *Univ Calif, Howard Hughes Med Inst, Dept Physiol, Box 0444, San Francisco, Ca 94143, USA. Genome-wide study of aging and oxidative stress response in *Drosophila melanogaster*. *Proc Natl Acad Sci U S A* **97:** (25) 13726.

### 15 Technological advances

Behlke MA, Dames SA, McDonald WH, Gould KL, Devor EJ, Walder JA. 2000. Integrated DNA Technol Inc, 1710 Commercial Pk, Coal-

Copyright © 2001 John Wiley & Sons, Ltd. 

ville, Ia 52241, USA. Use of high specific activity StarFire™ oligonucleotide probes to visualize low-abundance pre-mRNA splicing intermediates in *S. pombe*. *Biotechniques* **29:** (4) 892.
Ben-Dor A, Bruhn L, Friedman N, Nachman I, Schummer M, Yakhini Z. 2000. Agilent Labs, Chem & Biol Syst Dept, 3500 Deer Creek Rd, Palo Alto, Ca 94304, USA. Tissue classification with gene expression profiles. *J Comput Biol* **7:** (3-4) 559.
Brown TJ, Anthony RM. 2000. Guys & St Thomas Hosp Trust, Dept Microbiol, London SE1 7EH, England. The addition of low numbers of 3′ thymine bases can be used to improve the hybridization signal of oligonucleotides for use within arrays on nylon supports. *J Microbiol Methods* **42:** (2) 203.
Carninci P, Shibata Y, Hayatsu N, Sugahara Y, Shibata K, Itoh M, Konno H, Okazaki Y, Muramatsu M, Hayashizaki Y. 2000. RIKEN, Genome Explorat Res Grp, Tsukuba, Ibaraki, Japan. Normalization and subtraction of cap-trapper-selected cDNAs to prepare full-length cDNA libraries for rapid discovery of new genes. *Genome Res* **10:** (10) 1617.
Chechetkin VR, Turygin AY, Proudnikov DY, Prokopenko DV, Kirillov EV, Mirzabekov AD. 2000. Russian Acad Sci, Engelhardt Inst Mol Biol, 32 Vavilov Str, RU-117984 Moscow, Russia. Sequencing by hybridization with the genetic 6-mer oligonucleotide microarray: An advanced scheme for data processing. *J Biomol Struct Dyn* **18:** (1) 83.
Dai SM, Chen HH, Chang C, Riggs AD, Flanagan SD*. 2000. *City of Hope Natl Med Ctr, Beckman Res Inst, Div Neurosci, Duarte, Ca 91010, USA. Ligation-mediated PCR for quantitative *in vivo* footprinting. *Nat Biotechnol* **18:** (10) 1108.
Ferguson JA, Steemers FJ, Walt DR*. 2000. *Tufts Univ, Dept Chem, Medford, Ma 02155, USA. High-density fiber-optic DNA random microsphere array. *Anal Chem* **72:** (22) 5618.
Ferro M, Seigneurin-Berny D, Rolland N, Chapel A, Salvi D, Garin J, Joyard J*. 2000. *CEA PCV DBMS, Lab Chim Prot, 17 rue Martyrs, FR-38054 Grenoble 9, France. Organic solvent extraction as a versatile procedure to identify hydrophobic chloroplast membrane proteins. *Electrophoresis* **21:** (16) 3517.
Hacia JG, Edgemon K, Fang N, Mayer RA, Sudano D, Hunt N, Collins FS*. 2000. NIH/NHGRI, Bldg 49, Room 3A14, Bethesda, Md 20892, USA. Oligonucleotide microarray based detection of repetitive sequence changes. *Hum Mutat* **16:** (4) 354.
Hadd AG, Goard MP, Rank DR, Jovanovich SB*. 2000. *Amersham Pharm Biotech, Mol Dynam, 928 East Arques Ave, Sunnyvale, Ca 94086, USA. Sub-microliter DNA sequencing for capillary array electrophoresis. *J Chromatogr A* **894:** (1-2) 191.
He Y, Pang HM, Yeung ES*. 2000. *US/DOE, Ames Lab, Ames, Ia 50011, USA. Integrated electro-osmotically-driven on-line sample purification system for nanoliter DNA sequencing by capillary electrophoresis. *J Chromatogr A* **894:** (1-2) 179.
Herbert B, Righetti PG*. 2000. *Univ Verona, Dept Agr & Ind Biotechnol, Str Grazie 15, IT-37134 Verona, Italy. A turning point in proteome analysis: Sample prefractionation *via* multicompartment electrolyzers with isoelectric membranes. *Electrophoresis* **21:** (17) 3639.
Jiang GF, Harrison DJ*. 2000. *Univ Alberta, Dept Chem, Edmonton, Alberta, Canada T6G 2G2. mRNA isolation in a microfluidic device for eventual integration of cDNA library construction. *Analyst* **125:** (12) 2176.
Kane MD, Jatkoe TA, Stumpf CR, Lu J, Thomas JD, Madore SJ*. 2000. *Pfizer Global R&D, Dept Mol Biol Genomics, 2800 Plymouth Rd, Ann Arbor, Mi 48105, USA. Assessment of the sensitivity and specificity of oligonucleotide (50mer) microarrays. *Nucleic Acids Res* **28:** (22) 4552.
Kuklin A, Shams S, Shah S. 2000. 11150 West Olympic Bldg, Suite 1170, Los Angeles, Ca 90064, USA. High throughput screening of gene expression signatures. *Genetica* **108:** (1) 41.
Liu SM, Shivashankar GV, Sano T, Libchaber A. 2000. NEC Res Inst, 4 Independence Way, Princeton, NJ 08540, USA. Method for linking a synthesized protein to its mRNA-DNA complex. *Biotechniques* **29:** (4) 792.
Lonneborg A, Jensen M. 2000. Agric Univ Norway, Norwegian Forest Res Inst, Hogskoleveien 12, NO-1432 As, Norway. Reliable and reproducible method to extract high-quality RNA from plant tissues rich in secondary metabolites. *Biotechniques* **29:** (4) 714.
Lopez MF, Berggren K, Chernokalskaya E, Lazarev A, Robinson M, Patton WF. 2000. Proteome Syst Inc, 25 Ind Dr, Chelmsford, Ma 01824, USA. A comparison of silver stain and SYPRO Ruby Protein Gel Stain with respect to protein detection in two-dimensional gels and identification by peptide mass profiling. *Electrophoresis* **21:** (17) 3673.
Niwa M, Maruyama H, Fujimoto T, Dohi K, Maruyama IN*. 2000. *Scripps Clin & Res Inst, Dept Cell Biol, 10550 Nth Torrey Pines Rd, La Jolla, Ca 92037, USA. Affinity selection of cDNA libraries by lambda phage surface display. *Gene* **256:** (1-2) 229.
O'Brien JC, Stickney JT, Porter MD*. 2000. *US/DOE, Ames Lab, Microanalyt Instrumentat Ctr, Ames, Ia 50011, USA. Preparation and characterization of self-assembled double-stranded DNA (dsDNA) microarrays for protein:dsDNA screening using atomic force microscopy. *Langmuir* **16:** (24) 9559.
Pasquali C, Vilbois F, Curchod ML, Van Huijsduijnen RH, Arigoni F. 2000. SPRI, Chemin des Aulx 14, CH-1228 Geneva, Switzerland. Mapping and identification of protein-protein interactions by two-dimensional far-Western immunoblotting. *Electrophoresis* **21:** (16) 3357.
Pelizzari CA, Khodarev NN, Gupta N, Calvin DP, Weichselbaum RR. 2000. Univ Chicago, Dept Radiat & Cellular Oncol, 5841 Sth Maryland Ave, Chicago, Il 60637, USA. Quantitative analysis of DNA array autoradiographs. *Nucleic Acids Res* **28:** (22) 4577.
Penn SG, Rank DR*, Hanzel DK, Barker DL. 2000. *Molecular Dynamics Inc, Advanced Res Team, Sunnyvale, Ca, USA. Mining the human genome using microarrays of open reading frames. *Nat Genet* **26:** (3) 315.
Rozycka M, Collins N, Stratton MR, Wooster R*. 2000. *Inst Canc Res, Sect Mol Carcinogenesis, 15 Cotswold Rd, Sutton SM2 5NG, England. Rapid detection of DNA sequence variants by conformation-sensitive capillary electrophoresis. *Genomics* **70:** (1) 34.
Sabounchi-Schutt F, Astrom J, Olsson I, Eklund A, Grunewald J, Bjellqvist B*. 2000. *Amersham Pharmacia Biotech, Bjorkgatan 30, SE-75184 Uppsala, Sweden. An Immobiline DryStrip application method enabling high-capacity two-dimensional gel electrophoresis. *Electrophoresis* **21:** (17) 3649.
Sawada K, Doyu M, Tanaka F, Sobue G, Kato K. 2000. Nagoya Univ, Dept Neurol, 65 Tsurumai showa, Nagoya, Aichi 466 8550, Japan. Detection of triplet repeat expansion in the human genome by use of hybridization signal intensity. *Anal Biochem* **286:** (1) 59.
Sha RJ, Liu FR, Millar DP, Seeman NC*. 2000. NYU, Dept Chem, New York, NY 10003, USA. Atomic force microscopy of parallel DNA branched junction arrays. *Chem Biol* **7:** (9) 743.
Smith TPL, Godtel RA, Lee RT. 2000. USDA/ARS, US Meat Anim Res Ctr, Clay Center, Ne 68933, USA. PCR-based setup for high-throughput cDNA library sequencing on the ABI 3700™ automated DNA sequencer. *Biotechniques* **29:** (4) 698.
Spiro A, Lowe M*, Brown D. 2000. *Loyola Coll, Dept Phys, Baltimore, Md 21210, USA. A bead-based method for multiplexed identification and quantitation of DNA sequences using flow cytometry. *Appl Environ Microbiol* **66:** (10) 4258.
Strizhkov BN, Drobyshev AL, Mikhailovich VM, Mirzabekov AD*. 2000. *Argonne Natl Lab, Biochip Technol Ctr, 9700 Sth Cass Ave, Argonne Il 60439, USA. PCR amplification on a microarray of gel-immobilized oligonucleotides: Detection of bacterial toxin- and drug-resistant genes and their mutations. *Biotechniques* **29:** (4) 844.
Theodossiou I, Olander MA, Sondergaard M, Thomas ORT*. 2000. *Tech Univ Denmark, Dept Biotechnol, Bldg 223, DK-2800 Lyngby, Denmark. New expanded bed adsorbents for the recovery of DNA. *Biotechnol Lett* **22:** (24) 1929.
Tseng WL, Hsieh MM, Wang SJ, Chang HT*. 2000. *Natl Taiwan Univ, Dept Chem, Taipei 10764, Taiwan. Effect of ionic strength, pH and polymer concentration on the separation of DNA fragments in the presence of electroosmotic flow. *J Chromatogr A* **894:** (1-2) 219.
Von Eggeling F, Davies H, Lomas L, Fiedler W, Junker K, Claussen U, Ernst G. 2000. Inst Humangenet & Anthropol, Kollegiengasse 10, DE-07740 Jena, Germany. Tissue-specific microdissection coupled with ProteinChip® array technologies: Applications in cancer research. *Biotechniques* **29:** (5) 1066.
Voss T, Haberl P. 2000. Boehringer Ingelheim, Dr Boehringer Gasse 5-11, AU-1121 Vienna, Austria. Observations on the reproducibility and matching efficacy of two-dimensional electrophoresis gels: Consequences for comprehensive data analysis. *Electrophoresis* **21:** (16) 3345.
Yan JX, Harry RA, Spibey C, Dunn MJ. 2000. Harefield Hosp, Imperial Coll, Heart Sci Ctr, Harefield UB9 6JH, England. Postelectrophoretic staining of proteins separated by two-dimensional gel electrophoresis using SYPro dyes. *Electrophoresis* **21:** (17) 3657.
Yan JX, Wait R, Berkelman T, Harry RA, Westbrook JA, Wheeler CH,

Copyright © 2001 John Wiley & Sons, Ltd. 

Dunn MJ. 2000. Address as above. A modified silver staining protocol for visualization of proteins compatible with matrix-assisted laser desorption/ionization and electrospray ionization/mass spectrometry. *Electrophoresis* **21:** (17) 3666.

Zhan W, Wang TL, Li SFY*. 2000. *Natl Univ Singapore, Dept Chem, 10 Kent Ridge Crescent, SG-119260 Singapore, Rep Singapore. Derivatization, extraction and concentration of amino acids and peptides by using organic/aqueous phases in capillary electrophoresis with fluorescence detection. *Electrophoresis* **21:** (17) 3593.

### 16 Bioinformatics

Abril JF, Guigo R. 2000. Univ Pompeu Fabra, Inst Municipal Invest Med, Grp Recerca Informatica Med, C/ Dr Aiguader 80, ES-08003 Barcelona, Spain. gff2ps: Visualizing genomic annotations. *Bioinformatics* **16:** (8) 743.

Akutsu T, Miyano S, Kuhara S. 2000. Univ Tokyo, Ctr Human Genome, Minato ku, 4-6-1 Shirokanedai, Tokyo 108 8639, Japan. Inferring qualitative relations in genetic networks and metabolic pathways. *Bioinformatics* **16:** (8) 727.

Alves R, Savageau NA*. 2000. *Univ Michigan, Dept Microbiol & Immunol, Med Sci Bldg 2, Ann Arbor, Mi 48109, USA. Extending the method of mathematically controlled comparison to include numerical comparisons. *Bioinformatics* **16:** (9) 786.

Azuaje F. 2000. Univ Dublin, Trinity Coll, Artificial Intelligence Grp, Dublin 2, Rep Ireland. Interpretation of genome expression patterns: Computational challenges and opportunities. *IEEE Eng Med Biol Mag* **19:** (6) 119.

Bingham J, Sudarsanam S*. 2000. *SUGEN, 230 East Grand Ave, Sth San Francisco, Ca 94080, USA. Visualizing large hierarchical clusters in hyperbolic space. *Bioinformatics* **16:** (7) 660.

Cannarozzi G, Hallett MT*, Norberg J, Zhou X. 2000. *ETH Zentrum, Computat Biochem Res Grp, Zurich, Switzerland. A cross-comparison of a large dataset of genes. *Bioinformatics* **16:** (7) 654.

Cohen BA, Mitra RD, Hughes JD, Church GM*. 2000. *Harvard Univ, Dept Genet, Boston, Ma 02115, USA. A computational analysis of whole-genome expression data reveals chromosomal domains of gene expression. *Nat Genet* **26:** (2) 183.

Croft L, Beatson SA, Whitchurch CB, Huang B, Blakeley RL, Mattick JS*. 2000. *Univ Queensland, Ctr Funct & Appl Genomics Brisbane, Qld 4072, Australia. An interactive web-based *Pseudomonas aeruginosa* genome database: Discovery of new genes, pathways and structures. *Microbiology* **146:** (10) 2351.

Dhaeseleer P, Liang SD, Somogyi R. 2000. Univ New Mexico, Dept Comp Sci, Albuquerque, NM 87131, USA. Genetic network inference: From co-expression clustering to reverse engineering. *Bioinformatics* **16:** (8) 707.

Friedman N, Linial M, Nachman I, Peer D. 2000. Hebrew Univ Jerusalem, Sch Comp Sci & Engn, IL-91904 Jerusalem, Israel. Using Bayesian networks to analyze expression data. *J Comput Biol* **7:** (3-4) 601.

George RA, Heringa J. 2000. Natl Inst Med Res, MRC Div Math Biol, London NW7 1AA, England. The REPRO server: Finding protein internal sequence repeats through the Web. *Trends Biochem Sci* **25:** (10) 515.

Gnanasekaran TV, Peri S, Arockiasamy A, Krishnaswamy S*. 2000. *Madurai Kamaraj Univ, Bioinformat Ctr, IN-625021 Madurai, Tamil Nadu, India. Profiles from structure based sequence alignment of porins can identify β stranded integral membrane proteins. *Bioinformatics* **16:** (9) 839.

Haas SA, Beissbarth T, Rivals E, Krause A, Vingron M. 2000. Deutsch Krebsforschingszentrum, Dept Theoret Bioinformatics, Neuenheimer Feld 280, DE-69120 Heidelberg, Germany. GeneNest: Automated generation and visualization of gene indices. *Trends Genet* **16:** (11) 521.

Hart RK, Royyuru AK*, Stolovitzky G, Califano A. 2000. *IBM Corp, Computat Biol Ctr, POB 218, Yorktown Heights, NY 10598, USA. Systematic and fully automated identification of protein sequence patterns. *J Comput Biol* **7:** (3-4) 585.

Ho NC, Jia LB, Driscoll CC, Gutter EM, Francomano CA*. 2000. *NIH/NHGRI, Med Genet Branch, Bethesda, Md 20892, USA. A skeletal gene database. *J Bone Miner Res* **15:** (11) 2095.

Hollenberg LCL. 2000. Max-Planck-Inst Kernphys, Saupfer Checkweg 1, DE-69117 Heidelberg, Germany. Fast quantum search algorithms in protein sequence comparisons: Quantum bioinformatics. *Phys Rev E* **62:** (5 Pt B) 7532.

Jamison DC, Thomas JW, Green ED*. 2000. *NIH/Natl Human Genome Res Inst, Genome Technol Branch, Bethesda, Md 20892, USA. ComboScreen facilitates the multiplex hybridization-based screening of high-density clone arrays. *Bioinformatics* **16:** (8) 678.

Katti MV, Sakharkar MK, Ranjekar PK, Gupta VS*. 2000. *Natl Chem Lab, Div Biochem Sci, IN-441008 Pune, India. TRES: Comparative promoter sequence analysis. *Bioinformatics* **16:** (8) 739.

Kim J, Moriyama EN, Warr CG, Clyne PJ, Carlson JR. 2000. Yale Univ, Dept Ecol & Evolut Biol, New Haven, Ct 06520, USA. Identification of novel multi-transmembrane proteins from genomic databases using quasi-periodic structural properties. *Bioinformatics* **16:** (9) 767.

Kohlbacher O, Lenhol HP. 2000. Max-Planck-Inst Informatik, DE-66123 Saarbrucken, Germany. BALL: Rapid software prototyping in computational molecular biology. *Bioinformatics* **16:** (9) 815.

Kuffner R, Zimmer R*, Lengauer T. 2000. *German Natl Res Ctr Information Technol, Inst Algorithms & Sci Comp, Schloss Birlinghoven, DE-53754 St Augustin, Germany. Pathway analysis in metabolic databases *via* differential metabolic display (DMD). *Bioinformatics* **16:** (9) 825.

Lemkin PF, Thornwall GC, Walton KD, Hennighausen L. 2000. NCI, Frederick Canc Res & Dev Ctr, Lab Expt & Computat Biol, Frederick, Md 21702, USA. The Microarray Explorer tool for data mining of cDNA microarrays: Application for the mammary gland. *Nucleic Acids Res* **28:** (22) 4452.

Manduchi E, Grant GR, McKenzie SE, Overton GC, Surrey S, Stoeckert CJ. 2000. Univ Penn, Ctr Bioinformat, Philadelphia Pa 19104, USA. Generation of patterns from gene expression data by assigning confidence to differentially expressed genes. *Bioinformatics* **16:** (8) 685.

Margulies EH, Innis JW. 2000. Univ Michigan, Dept Human Genet, Ann Arbor, Mi 48109, USA. eSAGE: Managing and analysing data generated with Serial Analysis of Gene Expression (SAGE). *Bioinformatics* **16:** (7) 650.

Natale DA, Galperin MY*, Tatusov RL, Koonin EV. 2000. *NIH/Natl Lib Med, Natl Ctr Biotechnol Information, Bethesda, Md 20894, USA. Using the COG database to improve gene recognition in complete genomes. *Genetica* **108:** (1) 9.

Newman JRS, Wolf E, Kim PS*. 2000. *MIT, Whitehead Inst Biomed Res, Howard Hughes Med Inst, Dept Biol, 9 Cambridge Ctr, Cambridge Ma 02142, USA. A computationally directed screen identifying interacting coiled coils from *Saccharomyces cerevisiae*. *Proc Natl Acad Sci U S A* **97:** (24) 13203.

Ng PC, Henikoff JG, Henikoff S*. 2000. *Fred Hutchinson Canc Res Ctr, 1100 Fairview Ave Nth, Seattle, Wa 98109, USA. PHAT: A transmembrane-specific substitution matrix. *Bioinformatics* **16:** (9) 760.

Patterton HG, Graves S*. 2000. *POB 1024, Los Alamos, NM 87544, USA. DNAssist: The integrated editing and analysis of molecular biology sequences in Windows. *Bioinformatics* **16:** (7) 652.

Plewniak F, Thompson JD, Poch O. 2000. ULP, CNRS INSERM, Inst Genet & Biol Mol & Cellulaire, Struct Biol Lab, BP 163, FR-67404 Illkirch Graffenstaden, France. Ballast: Blast postprocessing based on locally conserved segments. *Bioinformatics* **16:** (9) 750.

Prokop M, Damborsky J, Koca J*. 2000. *Masaryk Univ, Lab Biomol Struct & Dynam, Kotlarska 2, CZ-61137 Brno, Czech Republic. TRITON: *In silico* construction of protein mutants and prediction of their activities. *Bioinformatics* **16:** (9) 845.

Reinert K, Stoye J*, Will T. 2000. *Canc Res Ctr, Neuenheimer Feld 280, DE-69120 Heidelberg, Germany. An iterative method for faster sum-of-pairs multiple sequence alignment. *Bioinformatics* **16:** (9) 808.

Rognes T, Seeberg E. 2000. Univ Oslo, Natl Hosp, Inst Med Microbiol, NO-0027 Oslo, Norway. Six-fold speed-up of Smith-Waterman sequence database searches using parallel processing on common microprocessors. *Bioinformatics* **16:** (8) 699.

Siew N, Elofsson A, Rychiewski L, Fischer D*. 2000. *Ben Gurion Univ Negev, Dept Bioinformat Comp Sci, IL-84105 Beer Sheva, Israel. MaxSub: An automated measure for the assessment of protein structure prediction quality. *Bioinformatics* **16:** (9) 776.

Taudien S, Rump A, Platzer M, Drescher B, Schattevoy R, Gloeckner G, Dette M, Baumgart C, Weber J, Menzel U, Rosenthal A. 2000. Inst Mol Biotechnol, Dept Genome Anal, Beutenbergstr 11, DE-07743 Jena, Germany. RUMMAGE: A high-throughput sequence annotation system. *Trends Genet* **16:** (11) 519.

Zien A, Ratsch G, Mika S, Scholkopf B, Lengauer T, Muller KR. 2000. GMD SCAI, Schloss Birlinghoven, DE-53754 St Augustin, Germany. Engineering support vector machine kernels that recognize translation initiation sites. *Bioinformatics* **16:** (9) 799.

Copyright © 2001 John Wiley & Sons, Ltd. 
